# Is there a role for ‘modified VAD’ in the treatment of multiple myeloma?

**DOI:** 10.3332/ecancer.2009.136

**Published:** 2009-06-04

**Authors:** A Agazzi, S Sammassimo, D Laszlo, SJ Liptrott, R Cascio, A Alietti, C Rabascio, P Mancuso, G Pruneri, G Martinelli

**Affiliations:** 1 Haematoncology Division, European Institute of Oncology, Via Ripamonti 435, Milan, 20141, Italy; 2 Anatomopathology Division, European Institute of Oncology, Via Ripamonti 435, Milan, 20141, Italy

## Abstract

VAD, (Vincristine, Doxorubicin and Dexamethasone) was initially proposed as a salvage therapy for myeloma patients in whom prior alkylating agent therapy failed, although in recent years VAD has been surpassed by novel combination therapies with new biological agents such as thalidomide (and its derivative, lenalidomide) and bortezomib. After the excellent results obtained by the novel agents, VAD can no longer be proposed in preparation to autologous transplantation, although there are still indications that VAD remains useful and clinically relevant in the initial treatment of symptomatic multiple myeloma.

## Background

Multiple myeloma (MM) is a tumour of mature isotype-switched plasma cells that accumulate in the bone marrow causing anaemia, hypercalcemia, bone lesions and renal impairment in at least 30% of cases [1].

Melphalan, the first alkylating agent, introduced in 1958, was later supplemented with prednisone to form the MP regimen [2–3]. It has been the mainstay of conventional therapy for several decades, although the response rate is no more than 50% with less than 5% CR and an overall survival (OS) not exceeding three years [4–6]. Melphalan is easy to use on an outpatient basis and has a relative low toxicity profile. However it should be used with caution in patients with renal failure [7] and should be avoided in patients who are candidates for stem cell collection and autologous stem cell transplantation (SCT) because of its myelotoxicity [8–9]. In order to improve the efficacy of MP regimen, other combination treatments have been explored.

VAD, (vincristine, doxorubicin and dexamethasone) was initially proposed as a salvage therapy for myeloma patients in whom prior alkylating agent therapy failed [10]. In comparison to MP, VAD presents several advantages including a more rapid response (maximum response obtained after two courses), no dose reduction required in cases of impaired renal function and, importantly, it does not influence the stem cell collection as it is not toxic for hematological precursors. Since 1989 this combination became the most widely used induction regimen in preparation for autologous transplantation [11–12]. According to SWOG criteria, the overall response rate was 84%, with 28% complete remission when used as first line treatment [11]. Successive studies on the efficacy of VAD estimated overall response rate between 50% and 60%, according to standard criteria of Bladé *et al* [13]. In recent years the popularity of VAD has been tempered by the promising preliminary results of new biological agents such as thalidomide (and its derivative, lenalidomide) and bortezomib and due to its haematological (granulocytopenia and thrombocytopenia) and non-haematological toxicity (neurotoxicity and impaired cardiac function). A further disadvantage of VAD is the risk of catheter-related infections and thrombosis. Infections are reported to be the main side effect of VAD and seem to be related to steroid and concomitant granulocytopenia: 30–35% of patients had infectious episodes in some institutions [14].

In order to decrease some of the most relevant toxicities of conventional VAD without the detrimental effect on efficacy, we proposed a less intensive VAD in preparation for intensified chemotherapy and subsequent autologous transplant.

## Methods

Between January 1997 to January 2007, a ‘modified VAD’ was given to 56 previously untreated multiple myeloma patients as induction therapy prior to autologous transplantation. (Table 1: Patients’ characteristics). Median age was 58 years (range 44–67); according to Durie and Salmon classification eight patients were stage I with an evident progression (I PD), nine patients were stage II and 39 patients were stage III. Monoclonal immunoglobulin subtypes were as follows: IgG in 28 patients (50%), IgA in 13 patients (23%), light chains in ten patients (18%) and five patients (9%) had a non-secretory myeloma. Out of the 56 patients, ten received only modified VAD while 46 patients went on to receive intensified high-dose chemotherapy supported by autologous stem cell transplantation (tandem transplant: 38 patients; single transplant: eight patients). The conditioning regimens are listed in Table 2. The response criteria were defined according to Bladé *et al* consensus guidelines [13]: a complete remission (CR) was defined as no detectable monoclonal component in serum immunofixation, no evidence of Bence-Jones paraprotein in the urine and less than 5% morphologically and phenotypically normal plasma cells in the bone marrow. A partial remission (PR) was defined as a decrease of at least 50% serum and urine M-protein, a very good partial remission (VGPR) as a decrease of 90% serum M-component in both serum and urine and a near complete remission (nCR) was obtained when no detectable M-protein is present on serum immunoelectrophoresis but with a positive immunofixation. No response was defined as stable disease or disease progression. The modified VAD schedule was delivered as follows: a continuous infusion of vincristine (0.4mg/sqm) and doxorubicin (9mg/sqm) on days 1–3, and dexamethasone 40mg per day intravenous on days 1–3 and then orally on days 4 and 5 of each cycle. The timing of steroid administration differed to that of the standard VAD regimen of oral administration on days 1–4, 9–12 and 17–21 [15–16].

The treatment cycles were repeated at four-weekly intervals with an average of four cycles (range 2–8). No antimicrobial prophylaxis was proposed.

The validation of the modified schedule in terms of efficacy and toxicity was verified by an interim analysis after the first ten consecutive patients.

## Results

The overall response rate (CR, nCR, VGPR, PR) was 60%: CR and nCR 10%, VGPR and PR 50%, SD 40% (Table 3: Response to treatment). These results confirmed those observed in the first ten consecutive patients investigated in the interim analysis. The response was independent of stage of disease and of the monoclonal immunoglobulin and light chain type.

Ten patients who received modified VAD alone were excluded from the intensification and subsequent SCT for the following reasons: hepatitis and a DVT in one patient after the first two cycles of modified VAD; failed stem mobilization in two patients who went on to alternative therapy; bilateral pneumonia in one patient after the second cycle and was considered at high risk of complications from successive intensification. One patient had an aggressive breast cancer progression (breast cancer diagnosed prior to myeloma) after mobilization and was shifted to the appropriate treatment; one patient had hepatic progression and received salvage therapy; one patient was lost to follow-up after mobilization; one patient did not accept transfusions. One responding patient (PR) was a candidate for transplantation but could not proceed due to a lung infection after high-dose cyclophosphamide for SC mobilization. One patient (nCR) was lost to follow-up after the first two modified VAD cycles.

Three patients (5%) developed pneumonia of unknown origin, which was resolved with antifungal and antimicrobial combination therapy in all cases. Only one serious reversible neurological event (paralytic ileus) occurred. Liver toxicity (grade 2 WHO) caused a delay of therapy and a subsequent dose reduction in one patient.

No patients developed grade 3 or 4 granulocytopenia and/or thrombocytopenia. Eight out of 56 patients (14%) developed transient granulocytopenia G1, and 12 patients (21%) thrombocytopenia G1. No cumulative haematological toxicity was observed. Importantly, the reduced-intensity schedule did not affect stem cell mobilization capability: the median value of collected CD34+cells in the 46 mobilized patients was 9.0x106/kg (range 2.8–45.6). In almost all cases (>90%) one leucapheretic procedure was enough for collection. The monoclonal plasma cell contamination in the leucapheresis product was not evaluated.

Nausea, fatigue, constipation, peripheral neuropathy and skin rashes occurred but they were mild (G1 WHO) and reversible in all cases. Cardiac function was monitored by echocardiography in the first 32 consecutively treated patients before and after modified VAD, all of which maintained the same level of cardiac function and so the remaining patients were not given echocardiograms.

Deep venous thrombosis was clinically evident and diagnosed by ultrasound in five patients (9%).

The patients who received only VAD showed a time to progression (TTP) of 6.8 months (range 2–18 months) while for the 46 intensified patients who obtained an overall response rate of 90% after transplant the TTP was 32 months (range 26–42).

## Discussion

Although in recent years VAD has been surpassed by novel combination therapies (Thal+Dex, Bortezomib+Thal+Dex, Bortezomib+Dex) as frontline strategies for multiple myeloma patients, standard treatment has not yet been identified. Nowadays in many European and North American cancer institutes VAD is still proposed as induction treatment in preparation for autologous SC transplantation. Certainly the need for a central venous catheter, G2 and G3 (WHO) granulocytopenia as well as the potential for cardiac toxicity have played an important role in drawing conclusions about VAD-related deaths. In order to overcome the severe side effects as a result of the regimen’s toxicity, we modified the standard VAD by reducing the duration of administration of the three components of the schedule as reported before above. We believe that a less intensive regimen could translate into a reduced toxicity profile but may also decrease the overall response rate. In our single institution experience with 56 previously untreated patients, an overall response rate of 60% was obtained, in comparison to 60–65% as reported in the historical data. The best response (2% CR + 4% nCR) occurred in 10% of the patients compared to 7% with a CR with standard VAD [17]. Importantly we obtained a very low side effect rate in comparison to the standard VAD schedule. Regarding haematological toxicity, no patient experienced G3–G4 (WHO) granulocytopenia and only 12% of the patients had G1 (WHO) transient intercycle granulocytopenia which did not cause a delay in therapy in any case. With standard VAD, G3 granulocytopenia was registered in 12% of patients [17], probably as a consequence of low-dose dexamethasone. No life-threatening infectious complications occurred during and after treatment with the modified VAD: pneumonia occurred in 5% of patients but was resolved with conventional antimicrobial therapy.

Only one serious reversible neurological event (paralytic ileus) was registered during the ultimate cycle of treatment in one patient, in contrast G3 (WHO) neuropathy is often observed during standard VAD.

We observed five thrombotic events (9% of patients) mostly related to the central venous catheter (three events), indicating that local mechanical trauma and not the thrombogenic potential of the chemotherapy was the cause of this complication (7% in the literature) [17].

Moreover our schedule seems to be safe in terms of cardiac function: LVEF was always within normal limits (>60%) in all the 32 patients given modified VAD.

Over the last ten years the debate has been ongoing about the best initial treatment for multiple myeloma in order to obtain the best response in preparation for dose intensification and transplant: CR, nCR and VGPR status prior to high-dose chemotherapy seems to correlate with better EFS and OS post-transplantation [18]. Novel thalidomide-based combination therapies (Thal/Dex [19], Thal/MP [20], Thal/Dex/PegLD [21], Lenalidomide-based: Lenalidomide/Dex [22], Claritromycin/Lenalidomide/Dex [23], Lenalidomide/MP [24] and bortezomib-based: Bortezomib/Dex [25], Bortezomib/DT-Pace [26], Bortezomib/Adria/Dex [27], Bortezomib/Thal/Dex [28], Bortezomib/MP [29]) represent a promising future as first line myeloma treatments. Preliminary results in pivotal studies state their superiority in terms of response rate (up to 95% with Bortezomib/Adriamycin/Dex). The favorable impact on stem cell mobilization would suggest a crucial role of these new combinations in the induction phase prior to the intensification therapy with single or tandem autologous transplantation. In this scenario, the role of VAD in preparation for autologous transplant is surely under discussion.

## Conclusions

After the excellent results obtained by the novel agents, VAD can no longer be proposed in preparation to autologous transplantation, although there are still indications that VAD remains useful and clinically relevant in the initial treatment of symptomatic MM [22].

The results of this small study demonstrate that ‘modified VAD’ is comparable to ‘classic VAD’ in terms of efficacy with a better safety profile. Further because of the favourable toxic profile of this less intensive VAD scheme, modified VAD could be considered as an alternative therapeutic option in the elderly if a large tumour burden is present and when a rapid response in symptomatic patients is needed.

Moreover the modified VAD could also play a role in relapsed disease, as indicated in the NCCN guidelines 2009 [30].

## Figures and Tables

**Table 1:**
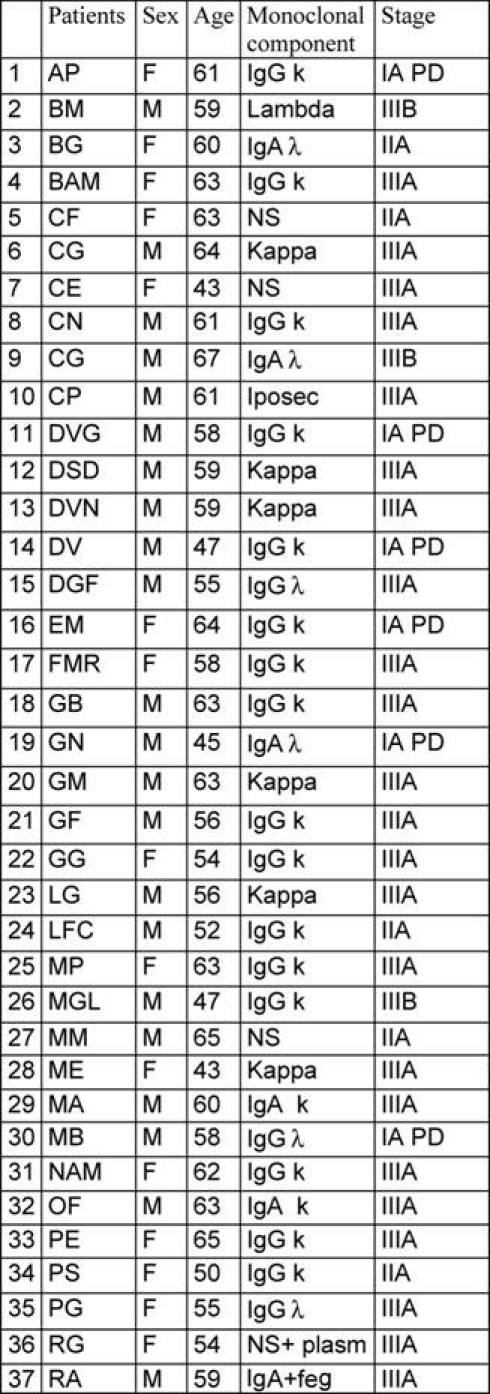
Patients’ characteristics

**Table 2:**
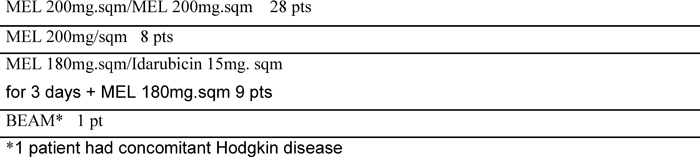
Conditioning regimens

**Table 3:**
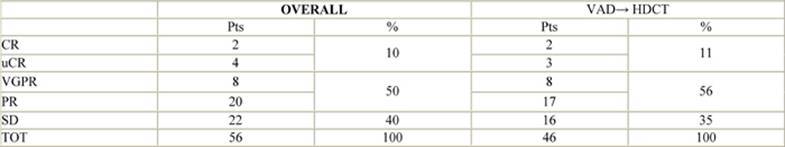
Response to modified VAD
